# Predicting risk of unplanned subsequent knee surgery following ACL reconstruction

**DOI:** 10.1186/s12891-026-09925-4

**Published:** 2026-07-02

**Authors:** Kathleen M. Poploski, Bicen Wang, Sahil Dadoo, Jeffrey B. Moorhead, Scott Rothenberger, Galen Switzer, Jonathan D. Hughes, Volker Musahl, Richard D. Boyce, James J. Irrgang

**Affiliations:** 1 Department of Physical Therapy, University of Pittsburgh, Pittsburgh, PA USA; 2 School of Computing and Information, University of Pittsburgh, Pennsylvania, USA; 3 Department of Orthopaedic Surgery, University of Pittsburgh, Pittsburgh, PA USA; 4 UPMC Freddie Fu Sports Medicine Center, Pittsburgh, PA USA; 5 University of Pittsburgh School of Medicine, Pittsburgh, PA USA; 6 Division of General Internal Medicine, University of Pittsburgh School of Medicine, Pittsburgh, PA USA; 7 Center for Research on Health Care Data Center, University of Pittsburgh School of Medicine, Pittsburgh, PA USA; 8 Department of Orthopaedics, University of Gothenburg, Gothenburg, Sweden; 9 Department of Biomedical Informatics, University of Pittsburgh, Pittsburgh, PA USA

**Keywords:** Revision, Risk factors, ACL reconstruction, Natural language processing

## Abstract

**Background:**

Following anterior cruciate ligament reconstruction (ACLR), unplanned subsequent knee surgeries are disruptive and costly. The purpose of this study was to identify risk factors associated with any subsequent knee surgery as well as specific surgical procedures, including revision, contralateral ACLR, surgery for meniscus injury, or procedure for loss of motion following primary ACLR using data from the electronic health record (EHR) of a large single-system health network.

**Methods:**

Data for individuals (≥14 years old) who underwent primary ACLR between January 2013 and June 2021 were extracted from the EHR. Potential patient and surgical predictors were identified from structured data and/or extracted from the ACLR operative report using natural language processing. Cox proportional hazards models were used to identify factors associated with the overall risk of subsequent knee surgery as well as specific surgical procedures (*p* < 0.05). Incidence per 100 person-years was reported.

**Results:**

From an initial sample of 6,121 individuals, 3,478 (median 21.8 years old [IQR 17.4, 32.9]; 44.2% female) underwent primary ACLR and were eligible for this study. The incidence rate per 100 person-years was 12.3 for any subsequent knee surgery, 2.7 for revision, 2.3 for contralateral ACLR, 4.3 for subsequent meniscus surgery, and 2.7 for subsequent procedure for loss of motion. No model was successful in identifying risk factors associated with any subsequent knee surgery. Risk factors associated with revision included younger age, medium surgeon volume compared with high volume (12–45 mean cases/year vs. ≥ 46), and sex as a time-varying coefficient (TVC), indicating females were initially at lower risk but risk increased with time. The only significant risk factor identified for subsequent contralateral ACLR was younger age as a TVC. Significant risk factors for subsequent meniscus surgery included younger age, medium surgeon volume compared with high volume, and medial meniscus repair at the time of primary ACLR. Risk factors for subsequent procedure for loss of motion included female sex, Black race, lateral meniscus repair, and quadriceps tendon autograft, while medial meniscectomy was found to be protective compared with no medial meniscus surgery.

**Conclusions:**

Risk factors varied by specific type of subsequent surgery following primary ACLR. Specifically, younger age was a risk factor for future ACL surgery, medium surgeon volume was associated with an increased risk of revision and subsequent meniscus surgery, medial meniscus repair was a risk factor for subsequent meniscus surgery, and lateral meniscus repair was a risk factor for a subsequent procedure for loss of motion. Consideration of risk factors relevant to subsequent surgeries beyond revision alone may better inform patient counseling prior to ACLR, as well as risk stratification and individualized post-operative rehabilitation following ACLR.

## Introduction

Studies on risk factors for revision after anterior cruciate ligament reconstruction (ACLR) have led to impactful changes in practice and the creation of clinical decision support resources [1, 2]. One large registry reported a 68% decrease in allograft use in patients ≤ 21 years old between 2010 and 2015, influenced by the dissemination of findings from their ACLR registry on the high rate of allograft rupture [3]. Several groups have also developed risk calculators for second ACL injury to guide shared-decision making for ACLR [1, 4]. 

While second ACLR, including revision of an ipsilateral ACLR and primary contralateral ACLR, is one of the most disabling unplanned subsequent knee surgeries, any surgery after ACLR is disruptive and costly. Yet, few studies have investigated risk factors specific to other unplanned subsequent knee surgeries, such as meniscus and loss of motion procedures, or the overall risk of returning to the operating room for an unplanned subsequent knee surgery. Consideration of risk factors for one type of unplanned subsequent knee surgery without other unplanned surgeries may provide an incomplete assessment of a patient’s overall risk of reoperation. As a result, it remains imperative to evaluate risk factors associated with all types of unplanned subsequent knee surgery within the same large cohort of patients undergoing primary ACLR.

Therefore, the purpose of this study was to identify risk factors associated with any unplanned subsequent knee surgery as well as specific procedures for revision, contralateral ACLR, meniscus injuries, or loss of motion following primary ACLR using electronic health record (EHR) data. It was hypothesized that patient-related and surgery-related factors would be associated with unplanned subsequent knee surgery and that factors would vary by the specific procedure. Important predictors of unplanned subsequent knee surgeries after ACLR may further inform impactful changes to patient management through shared decision-making, thereby enhancing clinical practice for the growing number of ACL injuries.

## Methods

### Dataset and overview

The Institutional Review Board at the University of Pittsburgh (STUDY21030078) reviewed and determined the study meets the regulatory requirements for exempt research under 45 CFR 46.104. Per the aforementioned institutional review board guidelines and review, informed consent to participate was not required from study participants. The data set for this project was obtained from the Health Record Research Request (R3) service of the Department of Biomedical Informatics [5]. Patients (≥ 14 years old) who underwent ACLR (Current Procedural Code [CPT]: 29888) within the UPMC Health Care System between January 2013 and June 2021 were identified. Patients who underwent ACLR (CPT: 29888) prior to 2013 at a UPMC facility were excluded from the data pull. For each patient, structured fields and unstructured clinical notes from the medical record were extracted from 2012 forward to identify relevant medical history prior to and following the primary ACLR.

Only patients with primary ACLR were included in the final analysis. Therefore, individuals who had a ACL revision, ACL repair, multiple ligament knee injury, injury requiring more extensive treatment, prior contralateral ACLR, or otherwise did not undergo primary ACLR as the index procedure were excluded (Appendix A) [6]. 

### Independent variables

The factors leading to unplanned subsequent knee surgery following ACLR are multifactorial and complex, therefore a comprehensive list of potential predictors was identified based on literature review, ongoing trials, clinical expertise, and availability in the dataset [1, 7–14]. Potential predictors available in the dataset included: (1) Patient factors (e.g., age, sex, body mass index [BMI], race, ethnicity) and (2) Surgical factors (e.g., procedures, anesthesia type, surgeon volume). Surgeon volume was calculated as the average number of ACLRs completed per surgeon per year active within the dataset. Patients were categorized as having undergone surgery by a high-volume surgeon (mean ≥ 46 mean ACLR cases per year), medium-volume (12–45 mean ACLR cases per year), or low-volume (< 12 mean ACLR cases per year, less than one per month). Some variables were extracted or calculated from structured data, including administrative codes (e.g., age, BMI, Functional Comorbidity Index (FCI) [15]), while most surgical factors (e.g., graft type, meniscal involvement) were extracted using natural language processing (NLP) pipelines developed and validated in Aim 1. 1Manual error analysis was used to adjudicate any conflicting cases resulting from the NLP extraction. See Appendix B and Appendix C for details of the data source used to identify, extract, and/or calculate each variable.

### Outcome

The overall outcome of interest for analysis was any unplanned subsequent knee surgery (referred to as “subsequent knee surgery” hereafter). Algorithms were developed and validated for identifying the most common classifications of subsequent knee surgeries, including revision ACLR, contralateral ACLR, and procedures for other knee ligaments, meniscus, cartilage, synovium, painful hardware, and loss of motion [see comment]. The algorithms used a combination of CPT codes, diagnoses codes, and/or keywords extracted from subsequent knee surgery operative reports to classify the procedure. These algorithms were used to identify individuals who underwent any subsequent knee surgery described above and specifically individuals who underwent a revision, contralateral ACLR, meniscal surgery, and/or procedure to recover loss of motion, as these were the most common subsequent surgeries. Revision included revision ACLR and other surgeries for graft failure, including bone-tunnel grafting and graft debridement. Subsequent procedures for loss of motion included manipulation under anesthesia, arthroscopic debridement, and/or arthrolysis of adhesions. Appendix D describes the details for inclusion and exclusion for each subsequent knee surgery analysis. For analysis of specific subsequent knee surgeries, individuals who did not have the outcome of interest but had another subsequent knee surgery were excluded. Patients who did not undergo subsequent knee surgery were censored at the last ortho-related encounter documentation within the EHR system (Appendix E) [16]. 

### Analysis

Descriptive statistics were calculated and reported as frequencies (%), means (standard deviation [SD]), and medians (interquartile range [IQR]) as appropriate. The overall rate of subsequent knee surgery and rate of each category of subsequent knee surgery was calculated as the proportion of individuals who underwent said subsequent knee surgery divided by the total number of individuals. Incidence rate, expressed as subsequent knee surgeries per 100 person-years, for each subsequent knee surgery type and overall were also calculated. Separate Cox proportional hazards models were fit to assess the association of each predictor with a binary overall subsequent knee surgery outcome and each specific subsequent knee surgery using backward elimination (p-value of > 0.1 for elimination). All models were adjusted for age and sex, regardless of significance and surgeon clustering [14]. Proportional hazards assumptions (log-log survival plots, Grambsch and Therneau analytical test method [17]), goodness-of-fit (Cox-Snell residual plot), and prediction ability (Harrel’s C-statistics) were assessed. If the proportional hazards assumption was violated for a predictor, that variable was evaluated as a time-varying covariate (TVC) and retained as a TVC if it remained significant. A time-varying coefficient allows the effects of a predictor on the hazard to change over time, rather than assume it remains constant. Final model results are presented as hazard ratios (HR) with 95% confidence intervals (CI). The final threshold for significance was set at α = 0.05. Stata (version 16.0, StataCorp, College Station, Texas, USA) was used for analysis.

## Results

**Fig. 1:**
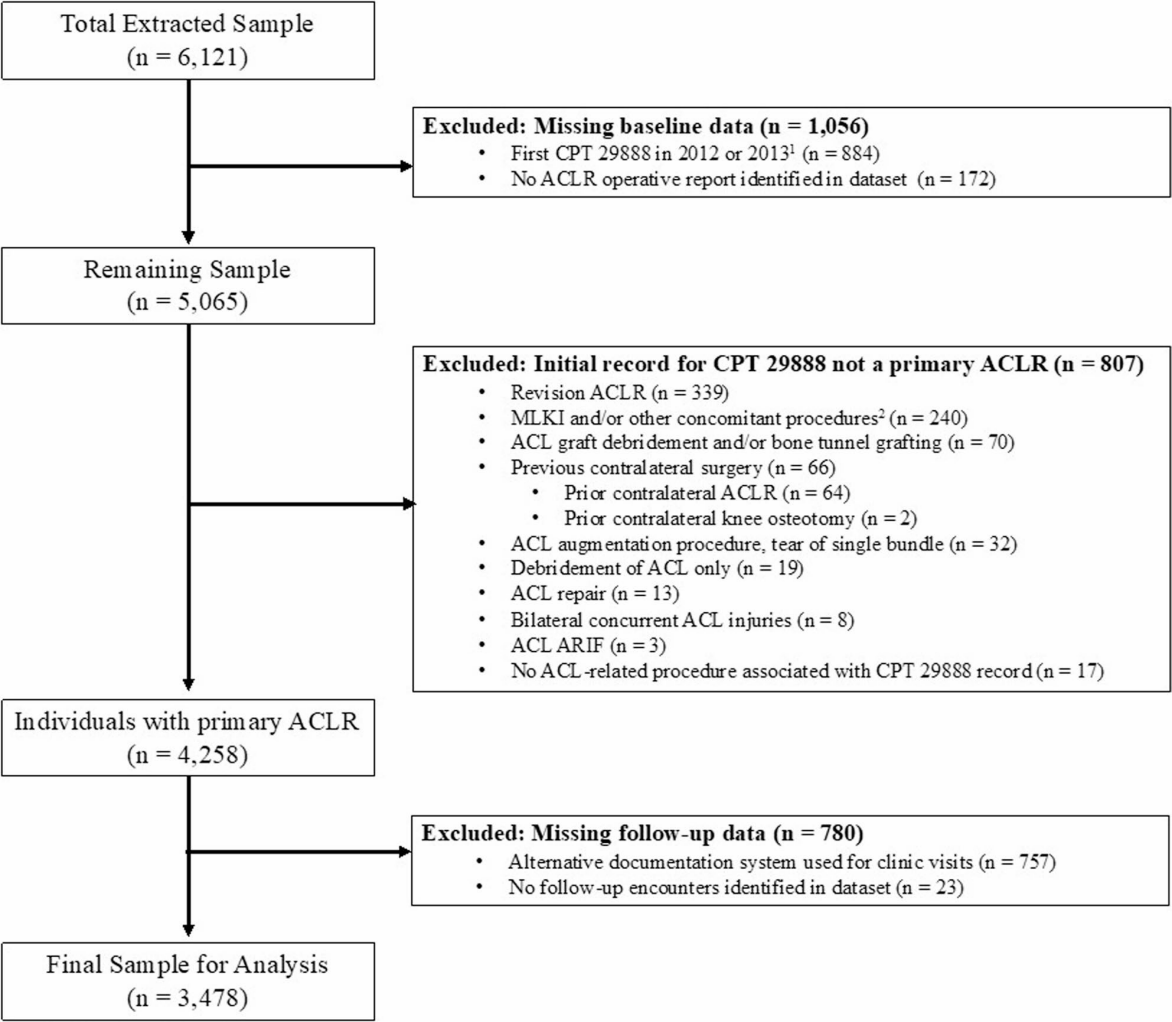
Flow Chart for Inclusion. ^1^ Cases were initially identified based on having record of CPT 29888 from 2013 forward. There were 82 cases with CPT 29888 in 2012 that were mistakenly extracted. There were 609 cases with CPT 29888 in 2013. These cases were dropped due to substantial missing data due to transition to EHR in 2013. ^2^ Examples of other concomitant procedures include procedures for fractures, osteotomy, and tendon repairs. This does not include procedures for meniscal or articular cartilage injury. ACLR, anterior cruciate ligament reconstruction; ARIF, arthroscopic reduction and internal fixation; CPT, Current Procedural Terminology; MLKI, multiple ligament knee injury

From an initial sample of 6,121 individuals, 1,056 individuals were excluded due to missing baseline data, 807 patients were excluded due to lack of primary ACLR as the index procedure, and 780 patients were excluded due to missing follow-up data (Fig. 1).

Therefore, 3,478 individuals (median 21.8 years old [IQR 17.4, 32.9], 44.2% female) underwent primary ACLR and were eligible for this study (Table 1).

**Table 1:** Demographics and Clinical Characteristics

	Primary ACLR
	(*n* = 3,478)
Age, median (IQR), years	21.8 (17.4, 32.9)
Female	1537 (44.2%)
Race	
White	2850 (81.9%)
Black	356 (10.2%)
Other	110 (3.2%)
Declined/missing	162 (4.7%)
Ethnicity	
Not Hispanic or Latino	3129 (90.0%)
Hispanic or Latino	49 (1.4%)
Declined/missing	300 (8.6%)
BMI, median (IQR), kg^2^/m	25.6 (22.8, 29.5)
< 18.5 (underweight)	34 (1.0%)
18.5 to < 25 (normal)	1303 (37.5%)
25 to < 30 (overweight)	956 (27.5%)
≥ 30 (obese)	689 (19.8%)
Missing	496 (14.3%)
Functional Comorbidity Index^a^	
mean ± sd [range]	0.7 ± 1.1 [0–8]
Prior knee surgery^b^	43 (1.2%)
Year of primary ACLR	
2014	478 (13.7%)
2015	436 (12.5%)
2016	493 (14.2%)
2017	471 (13.5%)
2018	481 (13.8%)
2019	519 (14.9%)
2020	363 (10.4%)
20,221 (January - June)	237 (6.8%)
Laterality	
Right	1723 (49.5)
ACL graft	
ACL autograft	2593 (74.6%)
BTB autograft	1372 (39.4%)
Hamstring tendon autograft	632 (18.2%)
Quadriceps tendon autograft	589 (16.9%)
ACL allograft^c^	885 (25.4%)
Achilles tendon allograft	81 (2.3%)
BTB allograft	151 (4.3%)
Hamstring tendon allograft	66 (1.9%)
Quadriceps tendon allograft	1 (0.0%)
Anterior tibial tendon allograft	230 (6.6%)
Posterior tibial tendon allograft	65 (1.9%)
Allograft, NOS	255 (7.3%)
ACL hybrid graft	36 (1.0%)
Medial meniscus procedure	1085 (31.2%)
Meniscectomy	450 (12.9%)
Repair^d^	635 (18.3%)
Lateral meniscus procedure	1159 (33.3%)
Meniscectomy	653 (18.8%)
Repair^e^	506 (14.5%)
Articular cartilage procedure	320 (9.2%)
Notchplasty	956 (27.5%)
Synovectomy	146 (4.2%)
Surgeon volume, mean cases/year^f^	
<12	559 (16.1%)
12–45	1075 (30.9%)
≥ 46	1844 (53.0%)

*ACLR * anterior cruciate ligament reconstruction, *BTB * bone patellar tendon bone, *NOS * not otherwise specified

^a^ The Functional Comorbidity Index is a comorbidity index designed to capture physical function. It assesses the presence (1) or absence (0) of 18 diagnosis, with a cumulative score ranging from 0–18.

^b^ Prior knee surgery includes knee surgeries which occurred in the year prior to initial ACLR; Meniscus surgery was the most common prior knee surgery (*n* = 34, 1.0%)

^c^ Hybrid grafts categorized with allografts for analysis

^d^ *n* = 19 individuals underwent medial meniscus repair and meniscectomy and are include with medial meniscal repair

^e ^*n* = 56 individuals underwent lateral meniscus repair and meniscectomy and are include with lateral meniscal repair

^f^ There were 7 surgeons included in the high-volume category (≥ 46 mean cases per year) with a range of 46.6–98.1 mean cases per year. There were 15 surgeons included in the medium volume category (12–45 mean cases per year) with a range of 11.7–35.2 cases per year). There were 20 surgeons included in the low volume category (< 12 mean cases per year) with a range of 1–11.0 cases per year)

The majority of the sample was White (81.9%) and non-Hispanic or Latino (90.0%). The median BMI was 25.6 (IQR 22.8–29.5) kg2/m, and most individuals had 0 (61.9%) or 1 (22.3%) comorbidity reported within 1 year of ACLR based on the FCI. 53% of individuals were treated by a high-volume surgeon (≥ 46 mean cases per year), 30.9% by a medium-volume surgeon (12–45 mean cases per year), and 16.1% by a low-volume surgeon (< 12 cases per year). Bone-patellar-tendon-bone (BTB) autograft (39.4%) was the most used graft, followed by hamstring tendon autograft (18.2%) and quadriceps tendon autografts (16.9%). Overall, allografts were used in 25.4% of cases, with anterior tibial tendon allograft (6.6%) being the most common specific type. The median follow-up time with an ortho-related encounter was 265 days (IQR 159–634), which equates to approximately 8.7 months.

There were 491 (14.1%) individuals who underwent at least 1 subsequent knee surgery, with an incidence of 12.3 subsequent knee surgeries per 100 person-years (Table 2). The incidence rate per 100 person-years was 2.7 for a subsequent surgery for revision, 2.3 for subsequent contralateral ACLR, 4.3 for subsequent meniscus surgery, and 2.7 for subsequent procedure for loss of motion. The shortest time to a subsequent knee surgery was for procedures for loss of motion which was performed at a median time of 119 days (IQR 83, 192) after ACLR, while the longest was for contralateral ACLR (715 days; IQR 399, 1028).

**Table 2:** Subsequent Knee Surgery Rates (*n* = 3,478)

	Overall rate *n*, (%)	Incidence Rate Subsequent surgeries per 100 person-years (95% CI)	Time to subsequent surgery Median (IQR), days
At least 1 subsequent surgery^a^	491(14.1%)	12.3 (11.3, 13.4)	425 (217–719)
At least 1 intra/extra-articular knee structure subsequent surgery^b^	376 (10.8%)	9.1 (8.2, 10.0)	540.5 (364, 805)
Ipsilateral			
Revision^c^	116 (3.3%)	2.7 (2.2, 3.2)	533.5 (379, 803)
Meniscus	180 (5.1%)	4.3 (3.7, 5.0)	530 (375, 773)
Loss of motion	113 (3.2%)	2.7 (2.2, 3.2)	119 (83, 192)
Contralateral			
ACLR	98 (2.8%)	2.3 (1.9, 2.8)	715 (399, 1028)

*ACLR * anterior cruciate ligament reconstruction

^a^ Includes revision and surgery for other knee ligament, meniscus, or cartilage, loss of motion, hardware removal, or fracture to the ipsilateral side or contralateral ACLR

^b^ Includes revision and surgery for other knee ligaments, meniscus, cartilage, fracture to the ipsilateral side or contralateral ACLR (Excludes cases of subsequent surgery for only loss of motion or hardware removal)

^c^ Includes revision ACLR and surgeries for graft failure including bone-tunnel grafting and graft debridement. There were 13 staged revisions, in which case time to first stage was used in analysis. There were 3 cases of graft failure where only procedures for bone-tunnel grafting or graft debridement were identified.

### Subsequent knee surgery

**Fig. 2:**
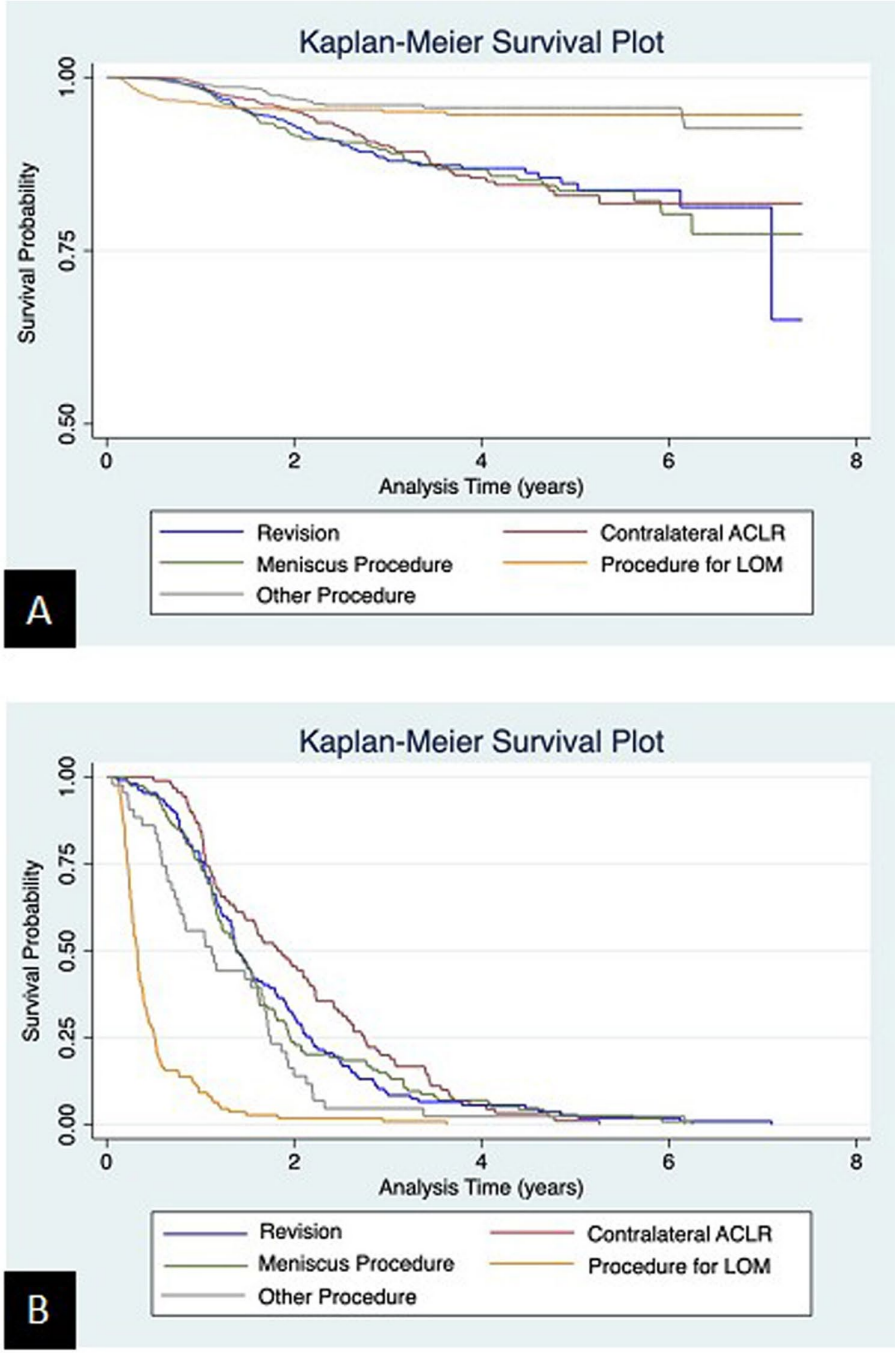
Survival Plots for Subsequent Knee Surgeries. **A **Kaplan-Meier Survival Plot for each subsequent knee surgery category. **B **Kaplan-Meier Survival Plots with only individuals who underwent subsequent surgery in each category highlighting differences in the time to subsequent procedure for loss of motion. ACLR, anterior cruciate ligament reconstruction; LOM, loss of motion

An overall model for risk of subsequent knee surgery was fit; however, it was highly reflective of predictors identified for subsequent procedure for loss of motion (described below) and when procedures for loss of motion were excluded, the selected variables were no longer significant. Given the unique survival trajectory of subsequent procedure for loss of motion (Fig. 2; median time to procedure of 119 days vs. > 530 days for other subsequent knee surgeries), the overall analysis was further refined to focus on procedures related to injury of intra or extra-articular structures (referred to as subsequent structural knee surgery hereafter) including revision, or surgical treatment of another ipsilateral knee ligament, meniscus or cartilage injuries, or fractures or contralateral ACLR, but excluding procedures for only loss of motion or hardware removal. The median time to subsequent structural knee surgery was 540.5 days (IQR 364, 805), and the incidence was 9.5 subsequent structural knee surgeries per 100 person-years. The final model to predict subsequent structural knee surgery included age, sex, medial meniscus procedure, and history of prior knee surgery; however, Harrell’s C-statistic was 0.51, indicating that the predictive discrimination was no better than chance. Therefore, only models for predictors of specific subsequent knee surgeries are presented (Table 3).

**Table 3:** Risk Factors for Subsequent Knee Surgeries

		HR	95% CI	*P* Value	Global *P* Value
Revision					
Main	Age	0.93	0.90–0.96	< 0.001	
	Sex, male as reference	0.54	0.33–0.86	0.010	
	Surgeon volume, high volume as reference (*≥* 46 case/year)				0.042
	<12 cases/year	1.24	0.61–2.53	0.558	
	12–45 cases/year	1.67	1.11–2.53	0.014	
TVC	Sex	2.14	1.17–3.92	0.014	
Subsequent contralateral ACLR					
Main	Age	0.96	0.91–1.00.91.00	0.067	
	Sex, male as reference	1.02	0.65–1.62	0.917	
TVC	Age	0.91	0.84–0.998	0.045	
Subsequent meniscus procedure					
Main	Age	0.96	0.94–0.98	< 0.001	
	Sex, male as reference	0.94	0.72–1.24	0.687	
	Surgeon volume, high volume as reference (*≥* 46 case/year)				0.052
	<12 cases/year	1.23	0.81–1.87	0.337	
	12–45 cases/year	1.34	1.06–1.71	0.015	
	Medial meniscus procedure, no procedure as reference				< 0.001
	Medial meniscectomy	1.08	0.61–1.92	0.792	
	Medial meniscus repair	2.48	1.95–3.16	< 0.001	
	Lateral meniscus procedure, no procedure as reference				0.202
	Lateral meniscectomy	1.15	0.74–1.79	0.524	
	Lateral meniscus repair	1.40	0.97–2.04	0.075	
Subsequent procedure for loss of motion					
Main	Age	0.99	0.96–1.02	0.648	
	Sex, male as reference	2.21	1.46–3.34	< 0.001	
	Race, white as reference				< 0.001
	Black	2.58	1.72–3.86	< 0.001	
	Other	0.68	0.24–1.97	0.48	
	Medial meniscus procedure, no procedure as reference				0.047
	Medial meniscectomy	0.43	0.21–0.85	0.015	
	Medial meniscus repair	0.87	0.61–1.23	0.422	
	Lateral meniscus procedure, no procedure as reference				0.001
	Lateral meniscectomy	0.97	0.51–1.85	0.930	
	Lateral meniscus repair	2.16	1.37–3.40	0.001	
	ACL graft type, BTB autograft as reference				0.001
	Hamstring tendon autograft	0.83	0.47–1.48	0.523	
	Quadriceps tendon autograft	1.94	1.05–3.59	0.033	
	Allograft	0.87	0.42–1.80	0.714	

*ACLR* anterior cruciate ligament reconstruction;* BTB* bone patellar tendon bone

### Revision

**Fig. 3:**
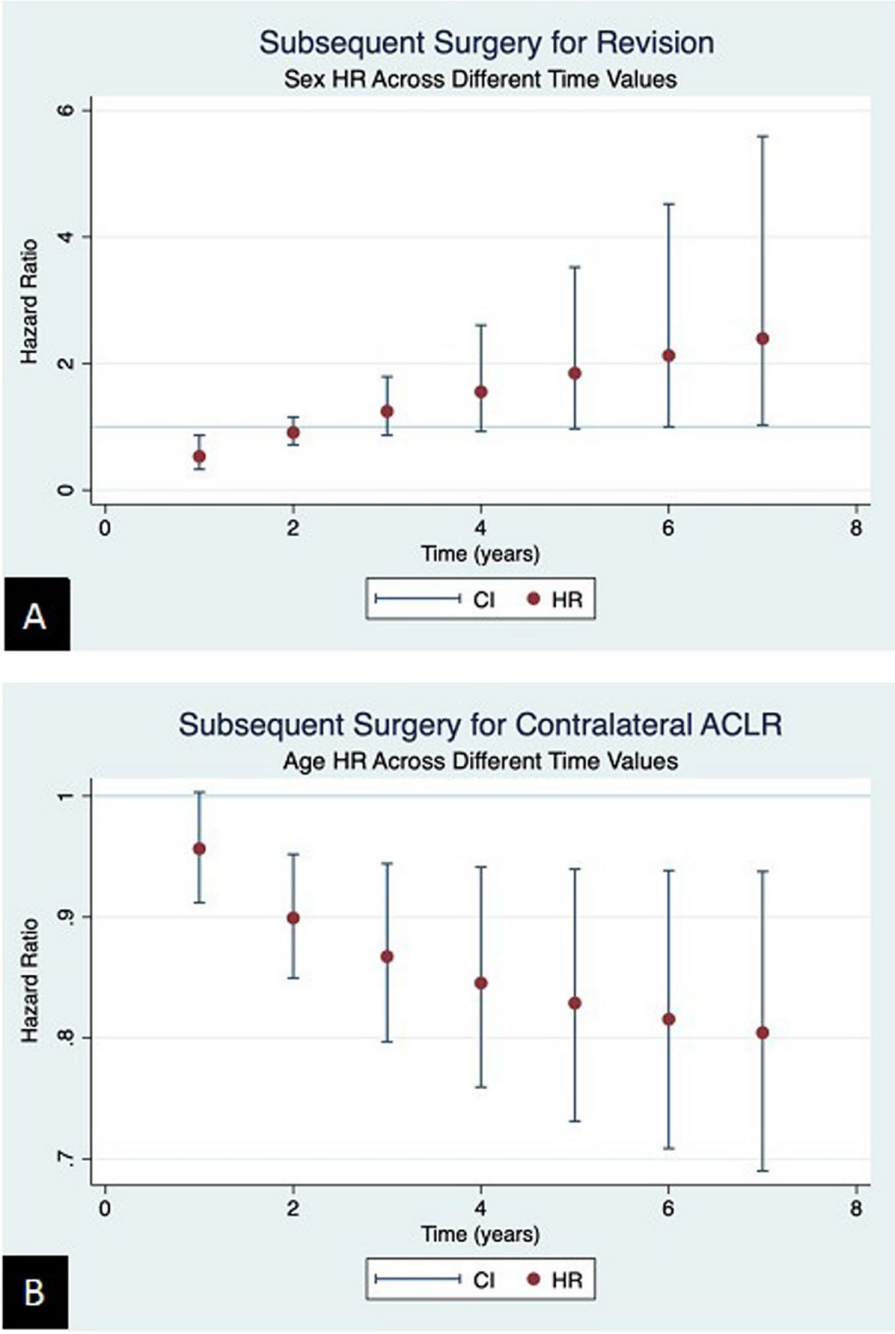
Plot of Hazard Ratios vs. Time for Time-Varying Coefficients. **A **Plot of Hazard Ratios for Sex vs. Time for Revision. Horizontal line indicates hazard ratio of 1. **B **Plot of Hazard Ratios for Age vs. Time for Contralateral ACLR. Horizontal line indicates hazard ratio of 1. ACLR, anterior cruciate ligament reconstruction; CI, Confidence Interval; HR, Hazard Ratio

The final variables selected for inclusion in the model for revision were age, sex, and surgeon volume. Sex did not meet the proportional hazards assumption and so was also included with a TVC. Each additional year of age was protective against revision (HR: 0.93; 95% CI: 0.90–0.96), while medium surgeon volume was associated with increased risk (HR: 1.67; 95% CI: 1.11–2.53) of revision compared with high surgeon volume. As shown in Fig. 3A, females were initially at lower risk of revision compared to males (HR: 0.54; 95% CI: 0.33–0.86); however, over time, the hazard ratio for sex increased. At approximately 2.5 years after primary ACLR, males and females were at equal risk, and then the risk for females compared to males continued to increase thereafter.

### Subsequent contralateral ACLR

The final variables included in the model for contralateral ACLR were age and sex. Age did not meet the proportional hazards assumption and so was also included with a TVC. The TVC of age was the only significant factor in the model. The baseline hazard ratio for age was 0.96 (95% CI: 0.91–1.00.91.00), and as shown in Fig. 3B, the hazard ratio associated with age decreased over time. This indicates that early on, age was not a significant factor related to contralateral ACLR, but further out from baseline surgery, each additional year of age was increasingly protective against contralateral ACLR.

### Subsequent meniscus surgery

The final variables selected for inclusion in the model for subsequent meniscus surgery were age, sex, surgeon volume, medial meniscus procedure, and lateral meniscus procedure. Each additional year of age was protective against subsequent meniscus surgery (HR 0.96; 95% CI:0.94–0.98) while medium surgeon volume (HR: 1.34; 95% CI:1.06–1.17) compared with high surgeon volume and medial meniscus repair compared with no meniscus surgery (HR: 2.48; 95% CI: 1.95–3.16) were significant risk factors.

### Subsequent procedure for loss of motion

The final variables selected for inclusion in the model for subsequent procedure for loss of motion were age, sex, race, medial meniscus procedure, lateral meniscus procedure, and graft type. Females were at increased risk compared to males (HR: 2.21; 95% CI:1.46–3.34), and Black individuals were at increased risk compared to White individuals (HR: 2.58; 95% CI:1.72–3.86). Quadriceps tendon autograft compared to BTB autograft was also a risk factor (HR: 1.94; 95% CI: 1.05–3.59). Medial meniscectomy vs. no medial meniscus procedure (HR: 0.43; 9% CI: 0.21–0.85) decreased risk of subsequent procedure for loss of motion, while lateral meniscus repair vs. no lateral meniscus procedure (HR: 2.16; 95% CI: 1.37–3.40) increased risk of subsequent procedure for loss of motion.

## Discussion

The most important finding of the present study was that risk factors varied by the specific type of subsequent knee surgery required following primary ACLR. Specifically, younger age was a risk factor for subsequent surgery for revision, meniscus procedures, and contralateral ACLR. Medium surgeon volume (12–45 mean cases per year) compared to high surgeon volume (≥ 46 mean cases per year) was associated with an increased risk of revision and subsequent meniscus surgery. Medial meniscus repair was a risk factor for subsequent meniscus surgery, and lateral meniscus repair was a risk factor for a subsequent procedure for loss of motion. Females were at increased risk of subsequent procedures for loss of motion but initially at lower risk of revision, though their risk increased over time.

Overall rates of subsequent knee surgery following ACLR reported in the literature vary substantially based on included surgeries, follow-up time, and reporting metrics. In this study, the overall incidence of subsequent knee surgery was 12.3 per 100 person-years with an overall rate of 14.1% of the included sample undergoing subsequent ipsilateral knee surgery or contralateral ACLR. While no single model was predictive of subsequent knee surgery in this study, younger age, allografts, female sex, concomitant knee surgery, and lower surgeon volume have all been associated with increased overall risk of subsequent knee surgery after ACLR in the literature, though the included subsequent surgeries and patient demographics vary from study to study [18, 19]. Comparison of incidence rates of subsequent knee surgery with major previous registry studies are summarized in Table 4.

**Table 4:** Comparison of Rates of Subsequent Knee Surgery with Previous Registry Studies

Registry-Based Outcomes:	ACLR Sample Size:	Mean Follow-Up Length:	Overall rate (%):	Incidence rate^a^:
Revision ACLR				
Kaiser Permanente ACLR	17,436	2.4 years	2.5%	1.1
Registry [1]				
New Zealand ACL Registry [20]	7,155	1.9 years	2.4%	1.3
MOON Study [12]	2,999	6 years (minimum)	7.5%	N/A
Present Study	3,478	8.7 months (median)	3.3%	2.7
Contralateral ACLR				
Kaiser Permanente ACLR	17,436	2.4 years	1.9%	0.8
Registry [1]				
New Zealand ACL Registry [21]	7,155	1.9 years	1.1%	N/A
MOON Study [11]	2,488	2 years (minimum)	3.5%	N/A
Present Study	3,478	8.7 months (median)	2.8%	2.3
Meniscus Surgery				
Kaiser Permanente ACLR	14,522	1.9 years	1.6%	1.1
Registry [14]				
New Zealand ACL Registry [22]	8,046	2.5 years	3.7%	N/A
MOON Study [12]	2,999	6 years (minimum)	11.9%	N/A
Present Study	3,478	8.7 months (median)	5.1%	4.3
Loss of Motion Surgery				
Kaiser Permanente ACLR	14,522	1.9 years	0.7%	0.4
Registry [14]				
New Zealand ACL Registry [22]	8,046	2.5 years	2.5%	N/A
MOON Study [12]	2,999	6 years (minimum)	7.8%	N/A
Present Study	3,478	8.7 months (median)	3.2%	2.7

^a ^Subsequent surgeries per 100 person-years

### Revision

The incidence per 100 person-years for revision was 2.7, which is higher than the rate of revisions reported for the Kaiser Permanente ACLR registry (1.1 per 100 person-years) [1], New Zealand ACL registry (1.3 per 100 person-years) [20], and other European registries (0.8–1.2 per 100 person-years) [23]. The difference in rates may be influenced by a number of factors, including that the median age of the current study was 21.8 years (IQR 17.4–32.9) compared with 27.2 years (IQR 18.7–37.7) in the Kaiser registry. Younger age has consistently been identified as a risk factor for revision [13]. Age is often a surrogate for activity level, and with each additional year of age, there may be less opportunity and/or desire to return to sport, which is one of the strongest risk factors for both revision and contralateral ACLR [24, 25]. Sex was also identified as a risk factor, with males initially at 87% greater risk compared to females; however, by 2.5 years after surgery, the risk of revision was equal between males and females and then continued to increase so that risk was greater for females compared males. There is conflicting evidence in the literature with regard to sex, with some studies reporting higher risk in males, others in females, and still others with no difference, which may be explained by a reversal in risk over time, as identified in this study [13]. 

There has also been differing information reported in the literature with regard to surgeon volume and risk of revision [26]. In this study, there was a 67% increased risk of subsequent surgery for revision for medium vs. high volume surgeons. Given the technical skill and expertise required for ACLR, it would be expected that high-volume surgeons would have decreased risk of subsequent surgery, though these surgeons may treat more complex cases, which should be considered in future studies.

### Subsequent contralateral ACLR

After controlling for sex, the TVC for age was the only significant factor associated with subsequent contralateral ACLR. As time passed, each additional year of age at the time of primary ACLR was associated with a lower risk of subsequent contralateral ACLR. For example, two years after ipsilateral ACLR, each additional year of age was associated with a 10% decrease in risk of subsequent contralateral ACLR, while at three years, it was associated with a 13% decrease in risk. Unfortunately, pre- or post-operative sports participation or activity level was unavailable in this dataset, but other studies have found higher rates of return to sport, and therefore exposure to high-risk activities, in younger individuals [27, 28]. Further research is needed to understand whether other potential age-related factors, including risk-taking behaviors or neuromuscular maturity, further explain the relationship between age and increased risk of subsequent ACL surgery [29]. 

### Subsequent meniscus surgery

In addition to age, medial meniscus repair and surgeon volume were associated with subsequent meniscal surgery. A number of studies have found meniscal repair at the time of primary ACLR to be a risk factor for subsequent meniscus surgery [14, 30], with three studies specifically finding an increased risk with medial meniscus repair [12, 31, 32]. Sullivan and Kim hypothesized that the increased risk with medial but not lateral meniscus repair may be due to the decreased mobility and increased biomechanical load to the medial meniscus, which may place higher stress on the repair, particularly when there is laxity or failure of the ACLR [12, 31]. Unfortunately, secondary osteoarthritis is associated with meniscectomy [33], suggesting that long-term benefits of repair need to be considered in light of shorter-term reoperation risk.

Having surgery by a high-volume surgeon performing nearly one ACLR per week (≥ 46 mean cases per year) compared with medium volume (12–45 mean cases per year) resulted in a 26% decrease in the risk of subsequent meniscus surgery. Lyman et al. similarly found that individuals who had surgery by a surgeon who performed at least 24 meniscal repairs annually were at lower risk of subsequent meniscal procedure [34]. Similar to other studies [35], high volume surgeons performed meniscal repairs more often than low or medium volume surgeons. In this study, high-volume surgeons performed meniscal repairs in 56% of cases of meniscal tear and meniscectomy in 30%. Low-volume surgeons performed meniscal repairs in 17% of cases with meniscal tear and meniscectomy in 69%, while medium-volume surgeons performed meniscal repair in 38% of cases with meniscal tear and meniscectomy in 43%.

### Subsequent procedure for loss of motion

The incidence rate in this study (2.7 per 100 person-years) was again higher than the Kaiser registry reported (0.4 per 100 person-years) [14]. A study by the MOON group reported that 7.8% of individuals in the cohort underwent a procedure for loss of motion within a 6-year follow-up [12]. Both of these studies also found females to be at higher risk of subsequent procedure for loss of motion [12, 14]. Females were at 148% (CI: 66% − 271%) increased risk of subsequent procedure for loss of motion in the Kaiser Registry study, comparable to the 121% (CI: 46–234%) increased risk found in the current study [14]. Black individuals were at 158% (CI 72–286%) increased risk for subsequent procedure for loss of motion compared to White individuals. This disparity likely reflects the impact of social determinants of health, including race, on outcomes following ACLR. Delays in access to care among Black patients may lead to more severe knee pathology at the time of initial ACLR [36], (see comments for citation) as well as barriers to post-operative and rehabilitative care services. These disparities in access to care should be further considered in relation to the risk of subsequent surgery.

Similar to the findings of this study, Huleatt and colleagues identified quadriceps tendon autograft and concomitant meniscal repair to be risk factors for manipulation under anesthesia and/or lysis of adhesions after ACLR [37]. In this study, 6.3% of individuals who had a quadriceps tendon autograft went on to have a procedure for loss of motion, similar to rates reported by Haley (7.2%) [38] and Huleatt (8.3%) [37] and higher than for rates found for BTB autograft (3.6%), hamstring tendon autograft (3.2%) or allograft (2.3%) in this study. Given the rise in popularity of quadriceps tendon autografts, this association is an important consideration for surgeons and physical therapists to be aware of during the rehabilitation process. Similarly, close attention to rehabilitation after meniscal repair is needed, as weight-bearing and range of motion are typically delayed to reduce shear forces and allow healing of the meniscus. The relationship between medial meniscectomy and decreased risk of subsequent procedure for loss of motion needs to be further explored in relation to those with and without meniscal injury.

### Clinical implications

There are several important clinical implications of the risk factors for subsequent knee surgery identified in this study. First, the time-dependent relationship for revision based on sex observed in this study may help to guide patient counseling and post-operative rehabilitation considerations. As discussed above, males initially were found to be at higher risk for revision in the early post-operative period, whereas females were found to have higher risk in the late post-operative period. Female sex is a well-known risk factor for ACLR failure and revision, with prior literature citing sex-specific anatomical differences, such as notch size, greater dynamic knee valgus during activities, greater ligament laxity, and hormonal influences as potential contributions [39, 40]. The higher risk among males for revision in the early post-operative period may reflect higher rates of, and earlier, return to sport compared to females, whereas the higher risk among females for revision in the long-term may reflect the above anatomical and biological considerations [41, 42]. While the exact etiology of primary ACLR failure was not examined in this study, males and females should be counseled separately about their risk for subsequent revision surgery. Additionally, post-operative rehabilitation protocols should include individualized return to sport testing to ensure appropriate timing of return to sport in males, while females may benefit from increased strength training for secondary stabilizers of the knee to reduce long-term risk of revision [43, 44]. 

In the present study, younger age patients were found to be at higher risk for both ipsilateral revision and contralateral ACLR. As discussed above, younger age is often a proxy for higher activity level, which inherently predisposes patients to higher risk of ACL injury [45]. In the younger patients who wish to return to sport, appropriate counseling on the long-term risk of both ipsilateral and contralateral ACL injury must be discussed. In addition, identifying addressable risk factors in these patients, such as hyperlaxity, at the time of primary ACLR is important to reduce the long-term risk of revision [46]. 

Medial meniscus repair was associated with higher risk of subsequent meniscus surgery, whereas lateral meniscus repair was associated with increased risk of subsequent surgery for loss of motion. Rehabilitation should be tailored to the specific structures involved and treated, particularly for the meniscus, with progression and restrictions adjusted accordingly to optimize healing and functional outcomes.

### Limitations

This study is unique in that NLP and EHR-based algorithms were used to develop the dataset. Overall, the findings of this study were consistent with the literature, lending credibility to the use of these methods to build EHR-based datasets more efficiently. The methods developed for this project can be used to build up-to-date risk prediction models for subsequent surgeries beyond revision and contralateral ACLR that can be used for shared decision-making to support individual patient management decisions. Using retrospective EHR data does come with limitations. This dataset was from a large health system offering care across the region, but patients may have moved out of the area or sought care elsewhere, leading to missed cases of subsequent knee surgery. A total of 780 individuals were excluded due to limited follow-up information leading to potential selection bias particularly given the over-representation flow (48.7%) and medium-volume (50.0%) surgeons in this excluded group(Appendix F) The median follow-up time with an ortho-related encounter was short (< 1 year), which may have led to an underestimation of events which often occur over longer timeframes (e.g., revisions and contralateral ALCR are often > 2 years after initial surgery). Other factors that likely influence outcomes or may potentially confound the relationships observed in this study, including factors related to rehabilitation, patient-reported measures, or specifics of the surgical procedure, (e.g., graft size or fixation method) were not able to be included in the study. Finally, while increasing literature suggests a higher posterior tibial slope may influence rates of ACL graft rupture and revision ACLR, radiographic variables were not assessed in the present study [47]. 

## Conclusion

Risk factors varied by specific type of subsequent surgery following primary ACLR.

Specifically, younger age was a risk factor for future ACL surgery, medium surgeon volume was associated with an increased risk of revision and subsequent meniscus surgery, medial meniscus repair was a risk factor for subsequent meniscus surgery, and lateral meniscus repair was a risk factor for a subsequent procedure for loss of motion. Consideration of risk factors relevant to subsequent surgeries beyond revision alone may better inform patient counseling prior to ACLR, as well as risk stratification and individualized post-operative rehabilitation following ACLR.

## Data Availability

The data that support the findings of this study are not publicly available due to reasons of sensitivity and to ensure patient confidentiality but it will be made available from corresponding author.

## Appendix

### Appendix A Codes and rules for exclusion from analysis

**Table:**

Method	Description/Codes	Note
First CPT 29888 in 2012 or 2013		Excluded all cases
Different documentation systems used for clinical visits	Identified by surgeons associated with private practice	Excluded all cases
Missing operative report associated with initial ACLR		Excluded all cases
Identified using NLP pipeline	Cases of revision, ACL debridement only, ACL graft debridement and/or tunnel grafting, ARIF, augmentation, ACL repair, bilateral ACL injury, MLKI, previous contralateral ACLR, no ACL procedure performed	Excluded all cases
Identified during manual chart review for the development of subsequent surgery identification algorithm (Aim 2)	Cases of revision, ACL debridement only, ACL graft debridement and/or tunnel grafting, ARIF, augmentation, ACL repair, bilateral ACL injury, MLKI, previous contralateral ACLR, no ACL procedure performed	Excluded all cases
CPT codes concomitant with initial ACLR (CPT 29888) indicating procedure for MLKI or other complex procedure	11012, 20694, 27025, 27305, 27360, 27385, 27405, 27409, 27418, 27420, 27422, 27425, 27427, 27428, 27429, 27450, 27457, 27487, 27498, 27524, 27535, 27536, 27538, 27580, 27602, 27652, 27658, 29850, 29851, 29855, 29871, 29873, 29889, 64708	Excluded all cases
CPT codes concomitant with initial ACLR (CPT 29888) indicating a potentially complex case	20670, 20680, 20900, 23700, 24101, 27299, 27310, 27331, 27332, 27333, 27403, 27407, 27448, 27450, 27455, 27457, 27570, 27705, 27707, 27709, 29805, 29855, 29884, 29914, 29916, 64704	Manually reviewed and excluded appropriate cases
CPT codes identified in the 1 year prior to ACLR	20670, 20680, 20690, 20693, 20694, 20900, 27332, 27333, 27340, 27345, 27347, 27350, 27380, 27381, 27385, 27386, 27403, 27405, 27407, 27409, 27412, 27415, 27416, 27418, 27420, 27422, 27424, 27425, 27427, 27428, 27429, 27435, 27448, 27450, 27455, 27457, 27508, 27509, 27510, 27511, 27513, 27514, 27520, 27524, 27530, 27532, 27535, 27536, 27538, 27540, 27550, 27552, 27556, 27557, 27558, 27560, 27562, 27566, 27570, 27599, 27705, 27707, 27709, 29850, 29851, 29855, 29866, 29867, 29868, 29873, 29874, 29875, 29876, 29877, 29879, 29880, 29881, 29882, 29883, 29884, 29885, 29886, 29887, 29889, G0289, G0428, S2112	Manually reviewed and excluded appropriate cases

*ACLR * anterior cruciate ligament reconstruction, *ARIF* arthroscopic reduction and internal fixation, *CPT* Current Procedural Terminology, *MLKI* multiple ligament knee injury, *NLP* natural language processing

### Appendix B Independent variables

**Table:**

Category	Independent Variable	Method for Identification
Patient	Sex	Structured data
	Age	Calculated from structured data
	Race	Structured data
	Ethnicity	Structured data
	BMI	Average of recorded BMIs within 3 months prior to ACLR
	Functional Comorbidity Index^a^	Calculated from ICD 9/10 diagnoses codes present within 1 year prior ACLR
	History of ipsilateral knee surgery (except ACLR)	Cases identified by CPT codes (Appendix C) present within 1 year prior to ACR. All manually reviewed and classified.
Surgery	Mean surgeon ACLR volume	Calculated as the average number of CPT 29888 associated with each surgeon per active year within the dataset
	Meniscal procedure	Identified from ACL operative report using NLP pipeline
	Cartilage procedure	Identified using algorithm for identifying cartilage procedure^b^
	Tourniquet use	Identified from ACL operative report using NLP pipeline^c^
	Notchplasty	Identified from ACL operative report using NLP pipeline^c^
	Synovectomy	Identified from ACL operative report using NLP pipeline^c^
	Graft type	Identified from ACL operative report using NLP pipeline^c^

*ACLR* anterior cruciate ligament reconstruction, *CPT* Current Procedural Terminology, *ICD* International Classification of Diseases, *NLP* natural language processing

^a ^The Functional Comorbidity Index is a comorbidity index designed to capture physical function. It assesses the presence (1) or absence (0) of 18 diagnoses, with a cumulative score ranging from 0-18.(15)

^b^Aim2

^c^Aim1

### Appendix C Codes used to identify cases with knee surgery prior to ACLR

**Table:**

Description	Code	Note
CPT codes identified in the 1 year prior to ACLR	20670, 20680, 20690, 20693, 20694, 20900, 27332, 27333, 27340, 27345, 27347, 27350, 27380, 27381, 27385, 27386, 27403, 27405, 27407, 27409, 27412, 27415, 27416, 27418, 27420, 27422, 27424, 27425, 27427, 27428, 27429, 27435, 27448, 27450, 27455, 27457, 27508, 27509, 27510, 27511, 27513, 27514, 27520, 27524, 27530, 27532, 27535, 27536, 27538, 27540, 27550, 27552, 27556, 27557, 27558, 27560, 27562, 27566, 27570, 27599, 27705, 27707, 27709, 29850, 29851, 29855, 29866, 29867, 29868, 29873, 29874, 29875, 29876, 29877, 29879, 29880, 29881, 29882, 29883, 29884, 29885, 29886, 29887, 29889, G0289, G0428, S2112	Manually reviewed and classified as appropriate

### Appendix D Criteria for subsequent surgery classification

**Table:**

Category	Criteria	Description
Any subsequent knee surgery	Inclusion	At least 1 subsequent knee surgery (revision, surgery for other knee ligament, meniscus, cartilage, loss of motion, hardware removal, fracture, or other) to the ipsilateral side or contralateral ACLR
Subsequent surgery for intra/extra-articular knee structure	Inclusion	At least 1 subsequent knee surgery to a native knee structure (surgery revision, other knee ligament, meniscus, cartilage, or fracture) to the ipsilateral side or contralateral ACLR.
	Exclusion	Excludes cases of subsequent surgery for only loss of motion or hardware removal)
Revision	Inclusion	Procedure for revision ACLR or graft debridement/bone tunnel grafting) at any subsequent surgery timepoint
	Exclusion	Subsequent contralateral ACLR prior to this procedure
Contralateral ACLR	Inclusion	Subsequent contralateral ACLR at any subsequent surgery timepoint
	Exclusion	Procedure for graft failure prior to this procedure
Meniscal surgery	Inclusion	Subsequent ipsilateral meniscal surgery, including cases concomitant with a procedure for graft failure
	Exclusion	Subsequent ACL procedure (ipsilateral failure or contralateral ACLR) prior to this procedure
Procedure for loss of motion	Inclusion	Subsequent ipsilateral procedure for loss of motion
	Exclusion	Subsequent ACL procedure (ipsilateral failure or contralateral ACLR) prior to this procedure

*ACLR* anterior cruciate ligament reconstruction

### Appendix E Criteria to determine follow-up time

**Table:**

Method	Description
Individuals with subsequent surgery	Date of initial subsequent surgery within relevant subsequent surgery category
Individuals with no subsequent surgery	Date of censoring identified based on the last ortho-related encounter documented within EHR system. This date was identified based on the 3 methods outlined below, with the last date used regardless of method of identification: - Last ortho encounter identified based on any encounter associated with an orthopedic surgeon who completed at least one ACLR within our dataset o Included encounter types: office visit, appointment, op report, CHP visit, outpatient, imaging, lab results, telemedicine, history, inpatient H&P, procedure visit, hospital-encounter, transcription, inpatient, IP consult, mobile encounter, hospital encounter, EKG, procedure note, IP lab results, testing visit - Last encounter where hospital service = ‘orthopedic surgery’ AND provider not null AND patient type not related to therapy - In Aim2 chart reviews, the date of last ortho clinic visit was recorded for each case reviewed. These dates were matched to the associated encounter type, encounter status, and encounter location in the dataset. These 3 criteria were then used to identify the last relevant encounter for each case

*ACLR* anterior cruciate ligament reconstruction, *EHR* electronic health record

### Appendix F Demographic and clinical characteristics including excluded cases

**Table:**

	Primary ACLR	Missing Follow-up	Overall(n = 4,258)
	(n = 3,478)	(n = 780)	
Age, mean ± sd (y)	26.3 ± 11.3	29.0 ± 12.2	26.8 ± 11.5
median (IQR)	21.8 (17.4, 32.9)	26.3 (18.3, 37.3)	22.2 (17.5, 33.9)
Sex			
Female	1537 (44.2%)	332 (42.6%)	1869 (43.9%)
Male	1941 (55.8%)	448 (57.4%)	2389 (56.1%)
Race			
White	2850 (81.9%)	654 (83.8%)	3504 (82.3%)
Black	356 (10.2%)	70 (9.0%)	426 (10.0%)
Other	110 (3.2%)	14 (1.8%)	124 (2.9%)
Declined/missing	162 (4.7%)	42 (5.4%)	204 (4.8%)
Ethnicity			
Not Hispanic or Latino	3129 (90.0%)	683 (87.6%)	3812 (89.5%)
Hispanic or Latino	49 (1.4%)	9 (1.2%)	58 (1.4%)
Declined/missing	300 (8.6%)	88 (11.3%)	388 (9.1%)
BMI, mean ± sd (kg^2^/m)	26.8 ± 5.5	29.2 ± 6.7	27.0 ± 5.7
median (IQR)	25.6 (22.8, 29.5)	27.5 (24.4, 33.4)	25.8 (23.0, 29.8)
< 18.5 (underweight)	34 (1.0%)	4 (0.5%)	38 (0.9%)
18.5 to < 25 (normal)	1303 (37.5%)	78 (10.0%)	1381 (32.4%)
25 to < 30 (overweight)	956 (27.5%)	109 (14.0%)	1065 (25.0%)
³ 30 (obese)	689 (19.8%)	103 (13.2%)	792 (18.6%)
Missing	496 (14.3%)	486 (62.3%)	982 (23.1%)
Functional Comorbidity Index^a^			
mean ± sd [range]	0.7 ± 1.1 [0-8]	0.8 ± 1.2 [0-7]	0.7 ± 1.1 [0-8]
Prior knee surgery^b^	43 (1.2%)	8 (1.0%)	51 (1.2%)
Year of primary ACLR			
2014	478 (13.7%)	149 (19.1%)	627 (14.7%)
2015	436 (12.5%)	128 (16.4%)	564 (13.2%)
2016	493 (14.2%)	118 (15.1%)	611 (14.3%)
2017	471 (13.5%)	104 (13.3%)	575 (13.5%)
2018	481 (13.8%)	83 (10.6%)	564 (13.2%)
2019	519 (14.9%)	84 (10.8%)	603 (14.2%)
2020	363 (10.4%)	67 (8.6%)	430 (10.1%)
20221 (January - June)	237 (6.8%)	47 (6.0%)	284 (6.7%)
Laterality			
Right	1723 (49.5%)	354 (45.4%)	2077 (48.8%)
Left	1755 (50.5%)	426 (54.6%)	2181 (51.2%)
ACL graft			
ACL autograft	2593 (74.6%)	473 (60.6%)	3066 (72.0%)
BTB autograft	1372 (39.4%)	316 (40.5%)	1688 (39.6%)
Hamstring autograft	632 (18.2%)	122 (15.6%)	754 (17.7%)
Quadriceps autograft	589 (16.9%)	35 (4.5%)	624 (14.7%)
ACL allograft^c^	885 (25.4%)	307 (39.4%)	1192 (28.0%)
Achilles allograft	81 (2.3%)	20 (2.6%)	101 (2.4%)
BTB allograft	151 (4.3%)	18 (2.3%)	169 (4.0%)
Hamstring allograft	66 (1.9%)	48 (6.2%)	114 (2.7%)
Quadriceps allograft	1 (0.0%)	1 (0.1%)	2 (0.0%)
Tibialis anterior allograft	230 (6.6%)	41 (5.3%)	271 (6.4%)
Tibialis posterior allograft	65 (1.9%)	112 (14.4%)	177 (4.2%)
Allograft, NOS	255 (7.3%)	52 (6.7%)	307 (7.2%)
ACL hybrid graft	36 (1.0%)	15 (1.9%)	51 (1.2%)
Medial meniscus procedure	1085 (31.2%)	313 (40.1%)	1398 (32.8%)
Meniscectomy	450 (12.9%)	181 (23.2%)	631 (14.8%)
Repair^d^	635 (18.3%)	132 (16.9%)	767 (18.0%)
Lateral meniscus procedure	1159 (33.3%)	301 (38.6%)	1460 (34.3%)
Meniscectomy	653 (18.8%)	222 (28.5%)	875 (20.5%)
Repair^e^	506 (14.5%)	79 (10.1%)	585 (13.7%)
Articular cartilage procedure	320 (9.2%)	119 (15.3%)	439 (10.3%)
Notchplasty	956 (27.5%)	335 (42.9%)	1291 (30.3%)
Synovectomy	146 (4.2%)	20 (2.6%)	166 (3.9%)
Tourniquet used	1765 (50.7%)	608 (77.9%)	2373 (55.7%)
Surgeon volume, mean cases/year			
<12	559 (16.1%)	380 (48.7%)	939 (22.1%)
12-45	1075 (30.9%)	390 (50.0%)	1465 (34.4%)
³ 46	1844 (53.0%)	10 (1.3%)	1854 (43.5%)

*ACLR* anterior cruciate ligament reconstruction, *BTB * bone patellar tendon bone, *NOS * not otherwise specified

^a^The Functional Comorbidity Index is a comorbidity index designed to capture physical function. It assesses the presence (1) or absence (0) of 18 diagnosis, with a cumulative score ranging from 0-18

^b^Prior knee surgery includes knee surgeries which occurred in the year prior to initial ACLR

^c^ Hybrid grafts categorized with allografts for analysis

^d^Cases of medial meniscectomy and medial meniscus repair included with the medial meniscus repair category

^e^Cases of lateral meniscectomy and lateral meniscus repair included with the lateral meniscus repair category

## Abbreviations

ACLR: Anterior cruciate ligament reconstruction
EHR: Electronic health record
CPT: Current procedural code
BMI: Body mass index
FCI: Functional comorbidity index
NLP: Natural language processing
SD: Standard deviation
IQR: Interquartile range
TVC: Time-varying covariate
HR: Hazard ratios
CI: Confidence intervals
BTB: Bone-patellar-tendon-bone

## Footnotes

1 Please note that Aim 1 is currently under review by the current journal as part of a three-project submission. As it is currently under review, we are unable to cite the manuscript, but hope to be able to in the future if accepted.
